# The role of freeze-dried plasma in a world of whole blood: a Canadian prehospital and transport perspective

**DOI:** 10.1186/s13049-026-01629-x

**Published:** 2026-05-14

**Authors:** Michael Peddle, Jan Trojanowski, Brodie Nolan

**Affiliations:** 1 Ornge, Mississauga, ON Canada; 2 Division of Emergency Medicine, Schulich School of Medicine, Western University, London, ON Canada; 3 British Columbia Emergency Health Services, Vancouver, BC Canada; 4 Department of Emergency Medicine, University of British Columbia, Vancouver, BC Canada; 5 Department of Emergency Medicine, St. Michael’s Hospital, Unity Health Toronto, 80 Bond Street, Toronto, ON M5B 1W8 Canada; 6 Division of Emergency Medicine, Department of Medicine, University of Toronto, Toronto, ON Canada

**Keywords:** Dried plasma, Transfusion, Hemorrhage, Trauma, Prehospital care, Emergency medical services

## Abstract

**Background:**

Hemorrhage is the leading preventable cause of trauma death, and timely transfusion remains a major challenge in Canada, particularly in rural and remote regions where long transport times create “blood deserts.” Whole blood has re-emerged as the gold standard for resuscitation, but donor limitations, short shelf-life, and cold-chain requirements restrict its universal deployment.

**Role of freeze-dried plasma:**

Freeze-dried plasma (FDP) offers a complementary solution with unique advantages for Canadian prehospital and transport systems. FDP is pathogen-reduced, shelf-stable for up to two years at room temperature, lightweight, and rapidly reconstituted at the point of care. Evidence from military and civilian studies demonstrates its logistical feasibility and safety, particularly in settings with prolonged transport times. FDP has been successfully integrated into NATO and Canadian Armed Forces operations and is increasingly recognized internationally as a practical adjunct to whole blood. In Canada, heterogeneous access to prehospital transfusion, high wastage rates of thawed plasma, and the inequities faced by Indigenous and remote communities highlight the urgent need for alternative strategies.

**Conclusion:**

FDP and whole blood are not competing but rather they are complementary therapies in a prehospital transfusion system. FDP represents a scalable, equitable, and operationally feasible option to extend balanced resuscitation to patients in rural and remote Canada. A coordinated national strategy, including regulatory approval, pilot projects, and outcome evaluation through networks such as CAN-PATT, is essential to move FDP from battlefield innovation to civilian standard of care.

Hemorrhage is the leading preventable cause of death in trauma and remains a major challenge in Canada, where vast geography and long transport times often delay access to life-saving transfusion [1, 2]. Early administration of blood and blood products has been shown to improve survival, yet patients in rural and remote frequently face “blood deserts,” with no access to blood transfusions in their immediate community [3]. In recent years, out-of-hospital transfusion programs have expanded across Canada, with most critical care transport organizations carrying red blood cells (RBCs) only, while some organizations also carry thawed plasma or prothrombin complex concentrate/fibrinogen concentrate to provide a more balanced ratio-based transfusion strategy [1, 4]. There is increasing interest in the adoption of whole blood in the prehospital environment, which provides balanced resuscitation in a single product [5, 6]. However, whole blood is constrained by donor availability, cold-chain requirements, and short shelf-life, limiting its reach outside of high-volume urban centres. Freeze-dried plasma (FDP) offers a complementary solution: it is pathogen-reduced, stable at room temperature for up to two years, lightweight, and rapidly reconstituted at the point of care [7–9]. Drawing on both military experience and emerging civilian evidence, this paper outlines the rationale for integrating FDP into Canada’s prehospital and transport systems as a practical and equitable adjunct to whole blood, particularly for rural and remote patients who remain furthest from definitive trauma care.

## Trauma, hemorrhage, and the need for early transfusion

Most deaths from hemorrhage occur within the first few hours after injury, underscoring the critical importance of early recognition and intervention. Traditional resuscitation strategies once relied heavily on crystalloid solutions; however, accumulating evidence has shown that large volume of crystalloid resuscitation is associated with worse outcomes and exacerbation of trauma-induced coagulopathy [10, 11]. This recognition has shifted modern practice toward damage control resuscitation, emphasizing early administration of blood products in balanced ratios that restore oxygen delivery and coagulation capacity simultaneously [12].

Randomized trials such as PAMPer and COMBAT, as well as pooled analyses, have demonstrated that the survival benefit of prehospital plasma is most apparent when transport times exceed 20 min [13–15], a scenario that reflects much of the Canadian geography. In rural and remote regions, patients often face transport delays of several hours, with no access to blood or blood components until arrival at a trauma centre. These prolonged intervals create “blood deserts” that place patients at heightened risk of preventable death. By establishing blood-on-board programs, Canadian critical care transport organizations can overcome these blood deserts by bringing the blood to the patient, as opposed to waiting to bring the patient to the blood [1, 4]. The Canadian Armed Forces have long recognized this challenge in deployed operations, leading to the adoption of both whole blood and freeze-dried plasma as early resuscitative products [16]. Translating these lessons into the civilian environment is essential to bridging the equity gap in trauma outcomes across Canada.

## Whole blood: the excitement and its limitations

Whole blood has re-emerged as a potential resuscitative product for patients with life-threatening hemorrhage due to trauma [5, 17–20]. By delivering RBCs, plasma, and platelets in physiologic proportions within a single unit, whole blood simplifies transfusion logistics and closely replicates the composition of circulating blood. Military and civilian studies have demonstrated that whole blood provides balanced hemostatic resuscitation, reduces the need for multiple product transfusions, and may improve survival in severely injured patients, however randomized clinical trial evidence is still lacking [17, 18, 21, 22]. In Canada, the Study of Whole Blood in Frontline Trauma (SWiFT Canada) is actively evaluating the feasibility and effectiveness of prehospital whole blood, and early implementation efforts highlight the enthusiasm for this strategy within critical care transport systems [6].

Despite these advantages, whole blood carries important limitations in the Canadian context. Maintaining a reliable supply depends on a sufficient donor pool, which is challenging given the need for male, type O donors with low-titer anti-A and anti-B antibodies. Whole blood must be stored at controlled temperatures and has a relatively short shelf-life of 21 days, with platelets becoming less functional over time. Additionally, currently Canada does not have procedures in place to separate the whole blood into individual components at the end of a unit’s lifespan. These constraints increase wastage, particularly in blood deserts as they are generally remote regions where utilization is infrequent. In addition, while whole blood is increasingly being studied and utilized for traumatic hemorrhage, its role in non-traumatic bleeding — such as obstetric, gastrointestinal, or vascular emergencies — is less well defined [5]. As a result, whole blood alone cannot serve as a universal solution across Canada’s diverse transport and rural care environments. Likewise, providing a balanced massive hemorrhage protocol with component therapy is equally unsustainable in the Canadian prehospital environment. The authors’ experiences with the SWiFT Canada study have shown wastage rates of thawed plasma have doubled since adding thawed plasma to the blood-on-board program (unpublished data). This is due to the short post-thaw shelf life seen with thawed plasma of only 5 days and limited hospital utilization opportunities despite being a tertiary site. It is anticipated even higher wastage rates of thawed plasma in remote, less busy blood-on-board locations if the use of thawed plasma were to expand and therefore will not be feasible from a system perspective. Addressing these gaps requires complementary strategies, with FDP offering a practical and scalable option to extend hemostatic resuscitation beyond the reach of whole blood programs.

## Freeze-dried plasma: rationale and evidence

Freeze-dried plasma is a thermostable formulation of human plasma that can be stored at room temperature for up to two years and reconstituted within minutes at the point of care [7, 8, 23]. Originally developed in Canada during World War II as dried serum, FDP has re-emerged as a safe and practical solution for modern trauma resuscitation. Advances in donor screening and pathogen inactivation have addressed the infectious risks that limited its historical use, and contemporary formulations demonstrate hemostatic properties equivalent to fresh frozen or thawed plasma [8]. These characteristics make FDP uniquely suited for austere environments, where refrigeration and continuous cold-chain storage are impractical or impossible.

Clinical evidence supports the operational feasibility of FDP in both military and civilian settings. The Canadian Armed Forces, NATO partners, and European civilian EMS systems have successfully deployed FDP to extend the “golden hour” by enabling earlier coagulation support in the field [7]. The PREHO-PLYO randomized trial in France demonstrated that prehospital lyophilized plasma transfusion is a feasible and safe procedure for patients who are at risk for hemorrhagic shock, although there was no evidence supporting its ability to prevent trauma-induced coagulopathy [24]. Additionally, the RePHILL trial in the United Kingdom demonstrated that FDP can be logistically integrated into prehospital trauma systems, however did not show that lyophilised plasma (LyoPlas) was superior to 0·9% sodium chloride for trauma related haemorrhagic shock in the civilian population studied [24, 25].

Beyond trauma, FDP may also play a role in non-traumatic hemorrhage, including obstetric and gastrointestinal bleeding, where early plasma administration could stabilize patients prior to definitive care. Operational testing under extreme conditions, including Arctic field studies, has confirmed FDP’s resilience to repeated freeze–thaw cycles and harsh environments [8]. Together, this evidence positions FDP not as a replacement for whole blood but as its essential complement, offering a flexible and scalable means to deliver hemostatic resuscitation in the Canadian out-of-hospital environment where geography, weather, and infrastructure limit access to traditional blood and blood components.

## Equity, access, and health system implications

The inequities in access to early transfusion are stark across Canada. Patients in urban centres may receive whole blood or component therapy within minutes of arrival at a trauma hospital, whereas those in rural and remote regions—particularly Indigenous communities—often wait hours before receiving any blood product. These delays contribute to disproportionate morbidity and mortality, reinforcing longstanding gaps in health outcomes tied to geography and systemic inequities. Addressing these disparities is both a clinical imperative and an issue of health equity.

FDP offers a practical pathway to narrow this divide. Its long shelf-life and stability at room temperature enable storage and use in locations where cold-chain infrastructure is unavailable, such as nursing stations, rural hospitals, and fixed- or rotary-wing aircraft. By providing earlier access to coagulation support, FDP can reduce reliance on crystalloids, mitigate trauma-induced coagulopathy, and improve survival odds for patients who would otherwise lack timely access to hemostatic resuscitation.

From a health system perspective, FDP also has the potential to improve efficiency and sustainability. Thawed plasma is associated with high wastage rates, particularly in low-volume transport bases where usage is unpredictable. FDP’s durability reduces product turnover and cost, while its ease of storage simplifies those logistics. Although unit costs are higher than conventional plasma, reduced wastage and operational savings may offset this difference over time.

The integration of FDP into Canada’s civilian trauma system requires regulatory approval, standardized protocols, and coordinated supply chains. Canadian Blood Services has also initiated domestic production efforts through spray-dried plasma technology, creating opportunities for another secure and scalable supply. Embedding FDP into national trauma protocols, with priority deployment to rural and Indigenous communities, represents an important step toward an equitable and resilient transfusion strategy for all Canadians.

## Research and implementation priorities

While FDP offers compelling advantages, its widespread adoption in Canada requires a deliberate and evidence-informed approach. Health Canada currently permits FDP (lyophilized, pooled donor, solvent detergent) use under the Special Access Program, for the Canadian Armed Forces. A fulsome Health Canada approval or Special Access approvals for interested critical care transport organizations are necessary for broader system integration. Additionally, there is increasing interest internationally in the use of single-donor, spray-dried plasma both in the military and civilian environments. It should be noted that a universal plasma product (e.g. AB plasma) would be required for routine prehospital use. Pilot projects in civilian critical care transport organizations and rural hospitals will be critical first steps to evaluate feasibility, safety, and cost-effectiveness in diverse operational contexts.

Integration of freeze-dried plasma into prehospital systems will require alignment with existing transfusion practices. Services should develop standardized reconstitution workflows and ensure appropriate training and competency for paramedics and transport clinicians. Temperature management and product warming should follow current blood transfusion protocols to mitigate hypothermia during resuscitation. As with other blood products, monitoring for transfusion reactions and clear documentation processes are essential. Finally, FDP should be incorporated into existing prehospital hemorrhage protocols as an early plasma component—either alongside red blood cells or when whole blood or thawed plasma is unavailable—to support timely hemostatic resuscitation in the prehospital environment.

A coordinated research agenda should focus on patient-centered outcomes, operational performance, and health system impact. Key areas include the effect of FDP on survival and morbidity in trauma patients with prolonged transport times; its role in non-traumatic hemorrhage such as obstetric or gastrointestinal bleeding; and its integration into prehospital hemorrhage protocols alongside whole blood and red blood cells. Equally important is the evaluation of wastage reduction and cost savings compared to thawed plasma, particularly in low-volume bases where plasma utilization is infrequent. National collaboration will be essential. The Canadian Prehospital and Transport Transfusion Network (CAN-PATT) provides a ready platform for data collection, knowledge translation, and development of standardized training modules for paramedics and transport physicians [1].

Finally, equity should remain a guiding principle for implementation. Deployment strategies should prioritize rural, remote, and Indigenous communities, where patients stand to benefit most from earlier coagulation support. Implementation in remote and Indigenous communities should occur in partnership with Indigenous leadership and local health authorities to ensure programs are culturally appropriate, responsive to community needs, and aligned with principles of equitable and respectful health system governance. By aligning regulatory approval, pilot projects, and coordinated data collection, Canada has the opportunity to move FDP from a battlefield innovation to a cornerstone of a civilian prehospital transfusion system that is both equitable and sustainable.

## Data Availability

Data sharing is not applicable to this article as no datasets were generated or analysed during the current study.

## Declarations

### About this supplement

This article has been published as part of Scandinavian Journal of Trauma, Resuscitation and Emergency Medicine Volume 34 Supplement 1, 2026: Proceedings of the Canadian Prehospital Transfusion Summit. The full contents of the supplement are available online at https://link.springer.com/journal/13049/volumes-and-issues/34-1/supplement.

## Abbreviations

FDP: Freeze-dried plasma
RBC: Red blood cells
SWiFT: Study of Whole blood in Frontline Trauma
CAN-PATT: Canadian Prehospital and Transport Transfusion Network
